# Development and trialling of a tool to support a systems approach to improve social determinants of health in rural and remote Australian communities: the healthy community assessment tool

**DOI:** 10.1186/1475-9276-12-15

**Published:** 2013-02-26

**Authors:** Elizabeth L McDonald, Ross Bailie, Thomas Michel

**Affiliations:** 1 Menzies School of Health Research, Charles Darwin University, Darwin, Australia

**Keywords:** Public health, Social determinants, Indigenous health, Remote, Environmental health

## Abstract

**Introduction:**

The residents of many Australian rural and remote communities do not have the essential infrastructure and services required to support healthy living conditions and community members choosing healthy lifestyle options. Improving these social determinants of health is seen to offer real opportunities to improve health among such disadvantaged populations. In this paper, we describe the development and trialling of a tool to measure, monitor and evaluate key social determinants of health at community level.

**Methods:**

The tool was developed and piloted through a multi-phase and iterative process that involved a series of consultations with community members and key stakeholders and trialling the tool in remote Indigenous communities in the Northern Territory of Australia.

**Results:**

The indicators were found to be robust, and by testing the tool on a number of different levels, face validity was confirmed. The scoring system was well understood and easily followed by Indigenous and non-Indigenous study participants. A facilitated small group process was found to reduce bias in scoring of indicators.

**Conclusion:**

The Healthy Community Assessment Tool offers a useful vehicle and process to help those involved in planning, service provision and more generally promoting improvements in community social determinants of health. The tool offers many potential uses and benefits for those seeking to address inequities in the social determinants of health in remote communities. Maximum benefits in using the tool are likely to be gained with cross-sector involvement and when assessments are part of a continuous quality improvement program.

## Introduction

Australians living in regional and remote areas experience poorer living conditions and health than people living in major cities. They have higher rates of death, disease, and higher levels of risk for chronic disease than those who live in major metropolitan areas [1]. The health of Indigenous (Aboriginal and Torres Strait Islander) peoples living in Australian remote and very remote communities is significantly worse than their counterparts living in urban centres and non-Indigenous Australians [2].

## Background

Prior to 1967, Governments in Australia largely overlooked the health and welfare needs of Indigenous peoples living in remote and rural Indigenous communities. From 1967, successive Federal, State and Territory Governments have introduced new policies to address the problem of the poor health and living conditions present in remote Indigenous communities. These policies have been described as confusing, disappointing, reactive and *ad hoc *[3-5]. Many have lead to serious unintended consequences that few were able to foresee. The Council of Australian Governments (COAG) has now agreed that all Governments (Federal, State and Territory) will work together to improve the social and economic wellbeing of Indigenous people and communities [6]. This new approach was necessary because COAG recognised that the significant commitment by Federal and State or Territory Governments to Indigenous issues is spread across many departments and agencies and results in a large number of programs that are often uncoordinated [7]. However, the Federal Government especially continues to fund and introduce single interventions to address the complex mix of social, cultural, political and economic factors that underlie poor health and the social problems present in these communities [8]. Policy initiatives such as the construction of new houses, and the introduction of the BasicsCard and store licensing, apparently perceived to have a ‘magic bullet’ effect to improve health and social outcomes among a population where extreme disadvantage persists. Federal and Territory Governments continue to provide new housing infrastructure without effectively addressing ongoing infrastructure repair and maintenance issues [8]. The primary reason given for the compulsory introduction of the BasicsCard for Indigenous social welfare recipients in the NT is to help families to manage their money to better meet essential household needs and expenses. A key objective of this income-management scheme is to restrict the amount of money that can be spent on ‘alcohol, tobacco and tobacco products, pornographic material, gambling products and services, gift cards, homebrew kits, or home-brew concentrates’ [9]. Few resources are directed at increasing community capacity and establishing effective community governance. Lack of timely investment means that major items of essential infrastructure in remote communities (constructed in the 1970s) are now failing. There is inequity between communities in regard to available infrastructure and access to programs and services [10]. There is concern that current Federal and Territory Government policy that focuses on providing new infrastructure (for example - housing, schools, stores, roads) and additional services only to designated ‘growth towns’ in the Northern Territory (NT) will promote further inequity [11,12]. Therefore, it remains that in many Australian rural and remote communities the essential infrastructure and services required to support people choosing healthy lifestyle options are missing. In these communities, people are disadvantaged on key social determinants of health [1,13]. Taking a systems approach to improving the social determinants of health is seen to offer real opportunities to improve health among such disadvantaged populations [14-16].

Taking action to improve social determinants of health is challenging because responsibility for these determinants is spread across a number of different government departments and agencies. In addition, the factors that influence improvements operate at a number of levels (national, regional and local), including policy, funding and operational levels. Currently there are few tools or mechanisms available to help assess, monitor and evaluate the appropriateness or effectiveness of government policies and services in addressing the social determinants of health in small rural and remote communities. Furthermore, community leaders do not have the information they require to advocate for the needs of their communities in a readily usable format. The Indigenous primary health care sector has successfully adapted continuous quality improvement (CQI) approaches to achieve incremental improvements in service delivery and health outcomes [17,18]. CQI methods have yet to be tested as a means to support efforts by government and non-government agencies to improve the social determinants of health in disadvantaged communities. Given Australian Government policies which purport to promote the better co-ordination of service delivery and ‘whole-of-government’ approaches to planning and service delivery in remote Indigenous communities [6], the lack of documented efforts to implement and evaluate systematic CQI methods in this context is remarkable. Development of the Health Community Assessment Tool (HCAT) is a step towards fostering a more comprehensive systematic approach to achieve incremental improvements in community infrastructure and services. In this paper, we describe the development of a tool that is designed to be used by a small group of community members or their representatives to measure, monitor and evaluate key social determinants of health in their community. We also provide findings pertaining to trialling the tool in four remote Aboriginal communities in the NT.

## Methods

The concepts and constructs that informed the development of the HCAT were drawn from a number of existing theories [19] and research findings [17,18,20-26]. Social ecological theory informs the general approach used in developing the tool. This theory provides a set of principles for understanding the interrelations among diverse personal and environmental factors in human health and illness [19]. The Driving Force, Pressure, State, Exposure, Effect (DPSEEA) framework and Multiple Exposure Multiple Effect Model informed how environmental health indicators might be used in this tool [27,28]. The DPSEEA framework defines driving forces (D) (for example -. housing policy), that lead to pressures on the environment (P) (for example – a shortage of housing or expensive rental cost), which in turn change the state of the environment (S) (example - overcrowding, resulting in human exposures (E1) (for example – increased transmission of infection, increased poor mental health, increased domestic violence), and thence to health effects (E2) (for example – respiratory disease or depression). Actions (A) can be taken at any point in this chain to mitigate or avoid unwanted health or social effects. Clearly, more upstream the action (at the level of the social determinates) the greater the likely benefit.

Our aim was to ensure that the tool would be: a) suitable for a range of Indigenous and non-Indigenous rural and remote community contexts; b) fit-for-purpose; c) user friendly; d) suitable to support potential CQI programs; and d) that it have the capacity to empower community leaders to take action to address inequities. The objectives of the trial were: a) to test the clarity and accuracy of the tool’s indicators by repeating the measurement of the same indicators in a community; b) to evaluate if the assessment of indicators varied markedly between participants and if so why; and c) to determine the appropriateness of the use of the tool in a facilitated small group discussion process. We aimed to assess tool reliability by calculating reliability coefficients but were aware that this would depend on the number of participants who would agree to take part in test and retest small group sessions and the characteristics of the participants.

### Study participants – tool development

To gain a range of perspectives from key stakeholders, groups made-up of Local and Territory Government and non-Government agency employees participated in HCAT design, development, and draft feedback and testing activities. Persons from a range of disciplines and those with experience working in key community positions were involved (for example, clinicians, allied health workers, public health officers, local government officers, housing officers, Aboriginal welfare officers). Individuals represented key government and non-government agencies, including the NT Government’s Departments of Health and Families (DHF), Department of Local Government, Housing and Sport, and Aboriginal and Medical Services Alliance NT. Key stakeholders from eight remote Aboriginal communities (four in the Top End of the NT and four in Central Australia) were invited and participated in the initial development stage.

### Study participants and setting – trial of the tool

For the final phase of community-based work, one NT Regional Shire Council nominated four communities under their administration to participate. These communities are located south-east of Darwin and all are classified as geographically remote using the Australian Institute of Health and Welfare Accessibility/Remoteness Index of Australia [29].

Ethics approval for the study was obtained from the Human Research Ethics Committees in the Top End of the NT and in Central Australia. Informed written consent was obtained from community peak bodies and individuals who participated in the study.

### Methodology

The tool was developed and piloted through a six phase and iterative process (described below) that involved consultation with community members and members of key stakeholder organisations at a number of points. A full description of the research leading to the tool development is the subject of another paper.

#### Literature review and tool development

Initially, our search of the literature focused on finding high-level evidence on environmental health indicators suitable for use in this context. Environment health defined in its broadest sense, that is comprising of those aspects of human health, disease, and injury that are determined or influenced by not only direct pathological effects of various chemical, physical, and biological agents, but also the effects on health of the broad physical and social environment [30].

**Figure 1:**
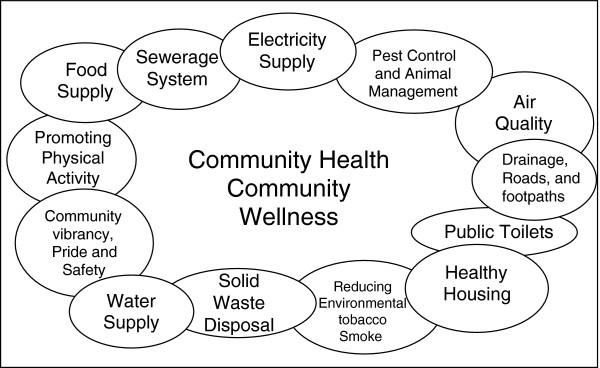
Infrastructure and programs considered important to promote good health and prevent chronic disease: key domains.

It soon became apparent that very little relevant evidence was available. It was necessary to draw on reports published by reputable Australian and international agencies to identify the range of domains to be covered by the tool and to determine ‘best practice’ in each of these domains. Identification of key domains (Figure 1) was based on: i) epidemiological evidence [1]; ii) components of infrastructure which are widely recognised as essential to promote health and prevent a wide range of diseases in remote community contexts, for example - water supply, sewerage system, food supply, waste disposal and housing [31-34]; and iii) common aspects of the community environment where inequities currently exist and where there is evidence that improved infrastructure and community programs can reduce the risk of developing chronic diseases, for example – providing opportunities for incidental and structured exercise (good drainage, footpaths, recreational facilities and programs) [32,35,36]; Tables 1 and 2 provide examples of domains, their components and associated indicators. The indicators are largely objective in their characteristics with descriptors of infrastructure and technology available in Indigenous and non-Indigenous rural and remote communities to guide scoring on each indicator.

**Table 1:** The healthy community assessment tool: Domain - water supply

	**Very poor**		**Poor**			**Satisfactory**			**Good**			**Excellent**
	**0**	**1**	**2**	**3**	**4**	**5**	**6**	**7**	**8**	**9**	**10**	**11**
1.1 Delivery system	Unprotected source, serious breakdowns with no preventative monitoring and maintenance program in place.		Basic monitoring and maintenance takes place but responsible local staff have poor skills and knowledge. There is no planned monitoring and maintenance program in place.			System well monitored and maintained. Local staff have satisfactory skills and knowledge. Water supply system can sometimes be maintained and serviced locally.			Protected source, system well monitored and maintained, responsible local staff have good skills and knowledge, water supply system can usually be maintained and serviced locally.			Well protected source. Formal monitoring and maintenance plan in place and in progress. Local responsible staff have excellent skills and knowledge and maintain and service the system.
1.2 Drinking water	Water quality does not meet ADWG values (e.g. microbiological failures occur or no regular disinfection process in place); boil water alerts may be regular.		Water quality almost meets ADWG values, some boil water alerts occur.			Water quality mostly meets ADWG values, i.e. occasionally some individual samples do not meet guidelines by marginal amounts. Drinking water is accessible and available to every house and at schools.			Water quality always meets ADWG values and drinking water is accessible and available to every house and at schools.			Water quality easily and consistently meets ADWG values. A water managementplan is in place. Drinking water is accessible and available in public places.
1.3 Rate of supply	System unable to provide quantity required for community all year round.		System consistently delivers at least the minimum water quantity requirements. Intermittent unplanned interruptions in supply occur.			System consistently delivers at least the minimum water quantity requirements. Intermittent planned interruptions in supply occur.			System consistently delivers at least the minimum water quantity requirements, no interruptions to supply.			System consistently delivers well above minimum water quantity requirements with no interruptions to supply. Supply plans take account of future needs.
1.4 Customer satisfaction	The water is considered to be unpalatable and unsafe. Rarely do community members drink water from the tap or other fixtures.		Sometimes the water is unpalatable. On occasion there is damage caused to fixtures or the water is discoloured. Regular complaints are experienced.			The water is generally palatable and considered to be healthy and safe. Individuals complain from time to time.			The water is palatable. Community members consider the water supply to be clean and healthy. No complaints.			Community members consider the water to be very pure andhealthy and superior in taste to other water supplies. No complaints.

**Table 2:** The healthy community assessment tool: Domain - pest control and animal management

	**Very poor**		**Poor**			**Satisfactory**			**Good**			**Excellent**
	**0**	**1**	**2**	**3**	**4**	**5**	**6**	**7**	**8**	**9**	**10**	**11**
8.1 Domestic pets	Uncontrolled breeding and large numbers of unwanted animals present. Damage to infrastructure and mess due to scavenging. Excessive noise from barking dogs. Dog faeces contaminate the environment. Regular complaints of dog bite. Community members feel unsafe walking in the community due to aggressive dogs.		Animal management strategy consists of the periodic culling of domestic animals.			Although in large numbers, pets are in a healthy condition and pose no threat to humans through disease or injury. Most animals are contained and few roam the community at large. Attempts made to restrict animals breeding. Complaints of dog bite infrequent.			Conditions apply as for ‘satisfactory’. Animal management programs and systems are in place, e.g. registration and desexing. Community members feel they can safely walk about in the community.			Conditions met as for ‘good’. In addition, a documented animal management plan is available and has been implemented. Progress towards achieving the objectives of the plan is monitored.
8.2 Livestock	Livestock roam free and are able to enter public and residential spaces. Livestock are in close proximity and pose a direct or indirect risk to community members through accident and injury and spread of infection (faeces, flies).		Livestock are routinely penned near houses and regularly escape and roam the community. The close presence of livestock is thought to contribute to the high number of flies in the community.			Livestock are healthy and well controlled and kept a safe distance away from the community.			Livestock are healthy, well managed and controlled and have designated areas well away from the community.			Conditions apply as for ‘good’. There is a proactive preventative vet program in place so livestock are routinely checked to promote their good health and prevent spread of disease.
8.3 Vermin	Infestations of pests and vermin are left untreated. The health of humans is directly at risks through bites and infestation. Vermin damage housing infrastructure including electrical wiring.		No community vermin or pest control program in place. Problems addressed on a house by house basis only. No action is taken to reduce the breeding of vermin in the community.			Community vermin and pest control program in place. Infestations of vermin are reported and addressed. Houses are ‘rat proofed’ and insect screens and other barriers against vermin are present.			Conditions apply as fir ‘satisfactory’. Action is taken to reduce breeding areas in the community. A monitoring program is in place to control the number of vermin. Houses are ‘rat proofed’. Insect screens and other barriers prevent vermin entering homes.			Conditions met as for ‘good’.Pest control systems readily accessible. Pest control chemicals are used and stored appropriately. Licensed pest control operators are used where appropriate. Community education programrunning to reduce numbers of vermin.

The tool was developed to offer a number of features. Each item is broken down into a number of components, for example, a ‘Water Supply’ system includes the hardware that forms the delivery system, water quality, and water pressure and customer satisfaction issues. The tool is intended to be educative and provide information to promote an understanding of how infrastructure and the community environment can support efforts to improve health. Information provided in the tool links issues about the need for appropriate programs to maximise any health and wellbeing benefits to be gained from infrastructure. It highlights the need to have programs to accompany infrastructure to maximise health and wellbeing benefits, for example - little benefit will be gained from a community centre that is not utilised by community members. The scoring system allows small improvements to be measured as only incremental improvements can be expected in some communities. This allows for ongoing monitoring and evaluation and use in a continuous quality improvement process. The tool also incorporates consideration of age and gender issues and inequities that may be present in communities (for example - that the elderly, girls and women have access to recreational programs and opportunities for increasing physical exercise).

#### Key stakeholder feedback

Key stakeholders and practitioners provided constructive feedback. They were able to draw the researchers’ attention to existing Australian Standards and policy documents of which the researchers were not aware. The draft tool was refined based on this new information.

#### Workshops

Through a process of two workshops, key Indigenous and non-Indigenous stakeholders worked in small multidisciplinary groups to evaluate the tool against set criteria. Criteria included: the degree to which indicators reflect real life circumstances in remote communities; indicators and their placement within the scoring scale; appropriateness and ease of use/interpretation of scoring scale; the tool’s overall level of readability.

In June 2008, workshops were held in Darwin and Alice Springs. The 2-day Darwin workshop was well attended (28–29 participants). Remote community representation at the workshop included seven Aboriginal Environmental Health Workers (AEHWs), one non-Indigenous community council chief executive officer, and one Aboriginal Community Services Officer. Professional disciplines represented included nutritionists, a physiotherapist, Environmental Health Officers (EHOs), AEHWs, Family and Children’s Services Workers, Health Promotion Officer, Housing Liaison Officers, Medical Officer and Remote Area Nurses. Key government and non-government agencies represented included the NT Government’s Departments of Health and Families (DHF) and Department of Local Government, Housing and Sport; Aboriginal and Medical Services Alliance NT, Menzies School of Health Research, and the Co-operative Research Centre for Aboriginal Health. Approximately 50% of participants were Indigenous and the majority were service providers living in remote communities or provided visiting services to these communities.

There were ten participants at the Alice Springs workshop. All but one participant was employed by the DHF. Disciplines represented at this workshop included eight EHOs, one nutritionist and one remote area nurse/researcher. Six Aboriginal participants from two remote communities had to cancel their attendance at short notice for urgent family reasons.

Workshop evaluation findings showed that participants considered that the subject and content of the draft tool was relevant to their work and reflected the issues they face in many remote communities. The scoring system was easily understood by both Indigenous and non-Indigenous participants and all felt comfortable with the small group process used to complete the assessment.

#### Expert review

The subsequent draft was sent to recognised ‘experts in the field’ (e.g. senior EHOs, nutritionist specialising in food supply systems to remote communities, engineers and administrators working in remote NT communities) for their review with further changes made based on this expert advice. Suggested changes from this group of reviewers were generally minor. An engineer who specialises in remote community water supply systems advised that a component be included in the domain “water supply” is consumer satisfaction. The nutritionist who specialises in remote community’s food supply systems provided information that lead to modifying indicators included in the domain “food supply”.

#### Community consultation

The researchers visited one community in each of four geographically dispersed NT health regions (Central, Barkly, Katherine and Top End) to consult with service providers and any interested community members about the purpose, content, and process for using the tool. These community visits revealed (and later confirmed during field testing) that low numeracy and literacy skills were not restricted to community members but also included many non-Indigenous persons employed to maintain essential community services in remote communities. This was highlighted during discussions when some Indigenous and non-Indigenous workers were very slow or had difficulty when the researchers asked them to read and review small amounts of information in the tool. This is opposed to their ease in understanding when the researchers read aloud the information contained in the tool.

#### Field testing

The final phase consisted of trialling the tool using a small group process in four remote communities. A small group facilitation protocol was developed to guide the small group process. This protocol takes into account issues such as facilitation and communication strategies for participants with English as a second language or those with poor numeracy and literacy levels. In addition, issues regarding cultural appropriateness and any perceived or real power imbalance between participants were taken into account, for example – gender issues and seating arrangements and the need for a neutral meeting venue. The facilitation role included multiple aspects, for example:

• The need to stay neutral and make sure all participants have a chance to speak;

• The need to reconcile the different viewpoints and lead the group to consensus;

• Monitoring of group dynamics, including if some individuals appear bored or inattentive, seem tense because of unvoiced disagreements, and/or appear frustrated because of the domination of one person or the length of time spent on one issue; and

• Ensuring the rate of discussion was acceptable to the participants, neither too fast nor too slow, and the meeting was completed in the allotted time.

An observer/note taker was seated in a non-intrusive position where discussion could be clearly heard and all group participants could be observed. The role of the observer included recording any issues that arose concerning:

• Indicator accuracy and any ambiguity in the written description of indicators; and

• Any issues that arose between participants and between participants and the facilitator.

At the completion of the meeting all participants were thanked and then asked how they found the small group process and if they had any suggestions of what might improve the process or content of the tool.

## Results

### Field-testing

Field-testing of the tool commenced in late 2009. Test and retest data was obtained in two of the four nominated communities. The sudden illness of the community-based local government administrator (known as the Shire Service Manager or SSM) in one community led to cancelation of activities in that community. In another community, the SSM appeared to be not fully supportive of the project and only the Aboriginal Essential Service Officer (AESO) was available to participate. Only initial test (Round 1) data was collected in this community. Test (Round 1) and retest (Round 2) data are available for two communities: Community A (population 580) and Community D (population 250). All participants were long-term residents employed directly by the Shire Council. In Community A, four participants took part in the Round 1 group session, while only one participant was available for the Round 2 session. In Community D, four participants took part in the Round 1 group session and four in the Round 2 session. One participant in each community took part in both Round 1 and 2 group sessions. Overall, the participants were predominantly Aboriginal men with only two Aboriginal women and one non-Indigenous male person participating.

In Communities A and D, 21 of the 42 indicators (50%) were scored the same in Rounds 1 and 2. The scores of a further six indicators (14-16%) scored within two points difference. Hence, there was good agreement (approximately 66%) between scores for the same indicator between Rounds 1 and 2 even when the make-up of the groups differed. In both communities, there was three or more points’ difference for 15 indicators.

In Community D, there were scoring discrepancies of 5, 6 and 7 points between the two rounds of data collection that could not be explained by changes in the level of services or condition of infrastructure because of new work. In this case, it was observed that for Round 1 the male, non-Indigenous SSM participant dominated discussion and he scored some indicators at an unrealistically high level. Other group members (including two women) remained silent and neither agreed or disagreed with these scores even though the facilitator provided them with opportunities to do so. It was clear that members of the group were not comfortable in contradicting this person. For Round 2 data collection the group included the same non-Indigenous SSM but also the community’s AESO and AEHW. These participants, taking the perspectives of both service provider and consumer, were well informed and more confident in disagreeing with the SSM’s views and modified his suggested scores. Hence, it would appear for Community D that the characteristics of the group and group dynamics were responsible for the discrepancies in scoring between Rounds 1 and 2 rather than any issue related to the tool.

In Community A, we observed two different reasons for discrepancies in scoring between rounds. Round 1 scoring appears to have been influenced by the characteristics of group members, and Round 2 scoring by the availability of only one participant. One participant in the Round 1 group was obviously very proud of his community’s achievements (the community had recently won a “Tidy Towns” award) and he largely went unchallenged by other group members in scoring some indicators at a high level. For Round 2, the AESO was the single participant and this participant scored the indicators that involved his own areas of work responsibility at a higher level.

### The participatory small group approach

All participants, including the one non-Indigenous participant, had low levels of education. English was the second language for all but the one non-Indigenous participant. These circumstances made it difficult for persons to read, comprehend and interpret written information quickly. The process for completing the tool included the facilitator:

• Providing a verbal description of the indicator that reflects the satisfactory state of a component;

• Asking the group if this description matches the state or condition of infrastructure or services in their community;

• After listening to the group’s discussion, and if required, verbally describing an indicator either higher or lower on the scale and asking if this is a better match;

• Managing the discussion until consensus is reached.

Facilitation proved appropriate for a number of reasons, including:

• Keeping participants engaged and the process moving along when some participants appeared to become bored;

• Avoiding a ‘one-size-fits-all’ approach. Indicators need to be applied to local contexts because, for example – communities’ water is drawn from various types of sources (bore, spring, river, mains) that require differing types of infrastructure, technology and maintenance regimes;

• Reassuring participants that the assessment was a ‘no-blame’ process and they should not feel threatened;

• Providing all participants with the opportunity to have input; and

• Providing the opportunity for participants to ask questions and clarify issues, adding an educative component to the process that facilitated informed decision-making.

Overall, the participants understood and related well to the indicators and their descriptions. The scoring system was very well understood and in all cases consensus was quickly reached. Participants made no recommendations about changing the small group process used or queried the descriptions of indicators or their level on the scoring scale.

All participants engaged at a high level and seemingly enjoyed discussions. The researchers perceived that group members viewed their participation as somewhat of a novelty event. Although the potential uses of the tool were explained, they perceived that neither Indigenous nor non-Indigenous participants clearly understood the potential benefits to their community in using the tool. This is not surprising given the day-to-day working atmosphere across communities is generally one of crisis. Indigenous and non-Indigenous community-level managers and workers across all sectors generally employ crisis management styles and have little time available for strategic thinking and planning. Furthermore, both Indigenous and non-Indigenous workers are generally ill equipped for the numerous and complex challenges they face in their positions in these communities. Their roles frequently do not include community development and strategic activities but have a focus on day-to-day service delivery.

## Discussion

We believe that the HCAT provides a useful vehicle and process to help those involved in planning, service provision, promoting equity within and between communities, and more generally promoting improvements in community social determinants of health taking a systems approach. Trialling the tool by repeating the measurement of the same indicators in a community showed the indicators to be readily understood and accurate, that is the indicators reflect the realities of many rural and remote communities. The assessment of some individual indicators did vary between participants but the risk of introducing bias into scoring can be countered by a) having an external facilitator who has a general knowledge of what infrastructure and services are available in a community; and b) having a small group rather than any one individual complete the assessment. The tool’s indicators rate well using an Indicator Rating Form developed specifically for examining indicators to be used in Indigenous communities [24]. Further work is underway to assess reliability using a small group facilitated assessment approach based on our experience to date. A funded research project that involves using the tool in 73 remote communities across the NT and Queensland will commence in 2013. Formal validity and reliability testing of indicators is part of this research. This testing will promote greater confidence in those using the tool.

There are several limitations of this research, including a lack of ‘evidence’ available to support some of the indicators and the limited range and number of participants involved in field-testing. However, our experience in using the tool outside of the research context, for example – for one-off community planning purposes, has shown that male and female Indigenous and non-Indigenous community members are happy to participate. The small number of participants, combined with different persons participating in Round 1 and 2 group sessions, meant it was not feasible to calculate reliability coefficients as intended. Some reliability testing is also possible through visual inspection, scientific testing and reviewing documentation. The tool should be used in conjunction with these methods to maximise validity of measurement and for educative purposes. Strengths of the tool include that it was developed using comprehensive review and feedback activities; the indicators are robust; the scoring system is anchored and objective in character; the tool was tested on a number of different levels and face validity was confirmed; and lastly the tool has been trialled at the community level.

Regional and central level managers of services and funders have shown a good level of interest in the tool since its development. The tool is currently being used for monitoring, evaluation and broader research purposes. This reflects emerging interest of key stakeholders and the current lack of tools to provide useful measures of environmental health and key social determinants of health in small rural and remote communities, or that are suitable for assessing, monitoring and evaluating the appropriateness or effectiveness of government policies and services as these affect remote Aboriginal communities. The NT Government’s EHOs now use the tool for assessment and planning purposes. The development of software to support a centralised data management system is now underway to maximise the use and availability of this data. Such a system will provide for automatic reporting on trends over time, and have the capacity to generate reports for each community, region and for the NT. Our attempts to get agreement from politicians and senior bureaucrats across sectors to support trialling the HCAT for the purpose of a ‘whole-of-government’ approach to remote community environmental health improvement has met with mixed responses. Short political cycles, rapidly changing political agendas, shifting policy priorities and vertical program models of funding provide obstacles to taking a more strategic approach to improving the social determinants of health in remote Aboriginal communities. Some of the challenges experienced in trying to achieve a whole-of-government approach in remote Aboriginal communities are described in a COAG trial evaluation report [7].

This comprehensive systems approach to community assessment, especially as part of a CQI program, should be helpful in overcoming the inequities in essential infrastructure and programs that rural and remote communities currently experience. The agencies that develop policies, fund, and provide services to remote communities often appear to operate in ‘silos’, and changing this silo approach continues to be challenging [37]. The HCAT offers a mechanism to help monitor and achieve stated Australian and NT Government policy aims of achieving better coordination between agencies and services, and the delivery of more appropriate, effective and efficient services [6].

## Conclusion

The HCAT is a prototype that offers many potential uses and benefits for community leaders, government officers and others seeking to measure environmental health conditions and address inequities in the social determinants of health in remote communities. Maximum benefits in using the tool will be gained when there is across sector involvement and assessments are part of a CQI program. Using the HCAT can highlight the critical requirement to address the social determinants of health that underlie poor health states and health related behaviour in remote Aboriginal communities.

## Abbreviations

AEHW: Aboriginal Environmental Health Worker; AESO: Aboriginal Essential Service Officer; COAG: Council of Australian Governments; CQI: Continuous Quality Improvement; DHF: Department of Health and Families; EHO: Environmental Health Officer; HCAT: Health Community Assessment Tool; NT: Northern Territory; SSM: Shire Service Manager

## Competing interests

The authors declare they have no competing interests.

## Authors’ contributions

EM conceived, designed and carried out the study and led the preparation of this paper. RB contributed towards designing the study and participated in the preparation of this paper. TM participated in all fieldwork activity, analysis of findings, and in the preparation of this paper. All authors read and approved the final manuscript.
